# Supplementary material for: Descriptive study of cattle and dog rabies cases in the Sheki-Zagatala region of Azerbaijan (2015–2016): Knowledge, attitudes, and practices of people towards rabies
Source: PLoS One. 2021 Nov 18;16(11):e0248572. doi: 10.1371/journal.pone.0248572 (PMC8601531; doi:10.1371/journal.pone.0248572)
Supplement: Attachment — Submitted filename: PONE_Review.docx [file pone.0248572.s001.docx]

1) The title of the study suggests a description of animal rabies cases. However, the study content suggests that this a study of knowledge about rabies among people who own certain animals. I strongly suggest the authors reframe the title of the study.

2) How many of the 84 animal rabies cases in two years (2015-2016) were cattle and dogs who died of rabies cases before Aug 14, 2016? If this number is >20, why have the authors included only 20 cases in this study?

3) Line 76 - Authors should mention "This study describes the epidemiological profile of animal rabies cases …….”. Why does the knowledge of owners about rabies not find mention in the study objectives???

4) In “Study design and data collection” section the preferable order of paragraphs seems Para 2, then Para 1, and then Para 3.

5) How were the non-cases selected? What is the rationale of interviewing non-cases? What information do they add to this study? Why did the authors choose 80 non-cases?

6) In “GPS Coordinates” section, the sentence seems incomplete. An “of” seems missing after “locations”.

7) In Table 1, it appears that only 4+1+1 = 6 dogs went out of the backyard, while in Line 120, the authors mention “8 were permitted to roam untethered in the community.”

8) What is meant by “Animal identification” in Table 1?

9) In Table 1, five dogs and seven cattle were reported to be bitten by ‘any animal’, but the biting animal adds to only 2 (1+1) for dogs and 6 (3+3) for cattle. Why is the information missing for others?

10) In Line 189, the authors mention that “residents in the Sheki-Zagatala region lack specific knowledge on rabies transmission, risk factors, and perceived severity of the disease”. There are several problems with this sentence. Firstly, the authors have not enquired about “severity of the disease” from the participants. Secondly, the very next sentence is “ …… correctly indicated as a means of transmission of the disease by a majority …….”. And more importantly, how is it justified to generalize the findings from 20+80=100 persons (method of selection unknown) to the entire population of the region?

11) Line 196: Recheck the first sentence. Did you intend to write “for” instead of “as”?

12) Line 206: The sentence “The locations of pastures ……” seems incomplete.

13) The “Discussion” section must contain a paragraph discussing the strengths and limitations of the study.
