# Supplementary material for: Descriptive study of cattle and dog rabies cases in the Sheki-Zagatala region of Azerbaijan (2015–2016): Knowledge, attitudes, and practices of people towards rabies
Source: PLoS One. 2021 Nov 18;16(11):e0248572. doi: 10.1371/journal.pone.0248572 (PMC8601531; doi:10.1371/journal.pone.0248572)
Supplement: Attachment — Submitted filename: PLOS One rabies in Azerbaijan_2015-2016_Review.docx [file pone.0248572.s002.docx]

This study describes the epidemiological situation of rabies in the Sheki-Zagatala region in Azerbaijan. In this region, despite the control measures implemented by the authorities, rabies cases still occurring in domestic and wild animals. The knowledge, attitude, and practice of residents were assessed. The results provide important information that will be used to improve the quality of current control measures to control rabies in the area.

However, some questions in this manuscript need to be clarified and improved before the acceptance for publication.

**Major comments:**

1. Look at the result when comparing the 20 cases and 80 no-case owners, it seems that the case owners have more knowledge, good attitude, and practices regarding rabies than the no-case owners. To confirm this observation, Statistical analysis should be applied to this data for comparison, which is not the case in this study; authors should think about it to give strong confidence and significance to the findings. In that case, a column can be created for each table to include the statistical values.

**Miner comments:**

1. Line 60-63: for the rabies situation in Azerbaijan some control measures such as vaccination was implemented and 465,000 animals were vaccinated between 2015 and 2016; 1,217,559 from 2012 to 2016. However, the total number of the animal population is not provided that can give an estimation of the vaccination coverage; and this data can be included in the discussion part to show whether the vaccination is still not effective to prevent rabies before speculating on other factors.
2. Line 102-104: the purpose of the electronic questionnaire is not clearly explained and data was not further exploited in the study (face to face interview was carried out); it can be deleted instead because not informative.
3. Line 106, the GPS coordinates can be included in the study design and data collection section, not as a subtitle.
4. Line 121-122: the respondents claimed that 3 vaccinated dogs were bitten but got infected and died, this information is not discussed in the study; this should be discussed as vaccine failure or incorrect information from the owners or died from other cause than rabies, ...
5. Line 128-130: It is stated that among 11 cattle, 3 were vaccinated against rabies for post-exposure; and 7 were bitten and died. Could the authors clarify if death cattle include 3 vaccinated ones? If so this should be discussed in the same way as for vaccinated dogs that died "vaccine failure or wrong information from owners, …”.
6. Given that the rabies cases in this study are based on clinical diagnosis, authors should briefly describe the clinical manifestations of rabies in different animal species that will support the results as provided in line 142-144 and figure 2. Description of furious and paralytic rabies can help readers to understand the situation, especially in line 161 where cattle bite humans.
7. Line 161 and Q3 in table 3: is cattle bite happened frequently in rabid cases? In figure 2, the aggressively against humans was reported only in 6 animals, are these cattle or dogs? Could you clarify this point?
8. Line 196-198: please include reference(s) for this statement about the common source and cause of rabies in Azerbaijan.
9. Line 206: remove by before the reference “ …was reported as a risk factor [15]"
10. Line 215: no information or data is revealing that rabies is underreported in the study, could you describe this point?
