# Supplementary material for: Descriptive study of cattle and dog rabies cases in the Sheki-Zagatala region of Azerbaijan (2015–2016): Knowledge, attitudes, and practices of people towards rabies
Source: PLoS One. 2021 Nov 18;16(11):e0248572. doi: 10.1371/journal.pone.0248572 (PMC8601531; doi:10.1371/journal.pone.0248572)
Supplement: Attachment — Submitted filename: Response to Reviewers.docx [file pone.0248572.s003.docx]

Simon Clegg, PhD	February 2, 2021

Academic Editor

PLOS ONE

Re: PONE-D-20-26983

Dear Dr. Clegg,

Thank you for inviting us to submit a revised draft of our manuscript now entitled “Descriptive study of cattle and dog rabies cases in the Sheki-Zagatala region of Azerbaijan (2015-2016): Knowledge, attitudes, and practices of people towards rabies in the region.” We also appreciate the time and effort you and each of the reviewers have dedicated to providing insightful feedback on ways to strengthen our paper. Thus, it is with great pleasure that we resubmit our article for further consideration. We have incorporated changes that reflect the detailed suggestions you have graciously provided. We also hope that our edits and the responses we provide below satisfactorily address all the issues and concerns you and the reviewers have noted.

To facilitate your review of our revisions, the following is a point-by-point response to the questions and comments delivered in your email dated Friday October 2, 2020.

**Editor’s Suggestions:**

1. Ensure manuscript meets PLOS ONE’s style requirements, including those for file naming

- Thank you for helping us ensure we have the correct formatting and style elements. We have updated the manuscript to ensure that it meets all style requirements for each section.

2. Table 4 was not referenced in the text of the manuscript

- Thank you for catching this. We discussed the table but failed to reference the table. We have updated the manuscript to include a reference to the table in our discussion (page 18, line 207 in Revised Manuscript with Track Changes).

3. The map contains images which may be copyrighted.

- Thank you for helping us understand this issue and for the helpful tips on developing a new map. We have generated a new map using an open-source tool from OpenStreetMap®. Documentation is licensed under the Creative Commons Attribution-ShareAlike 2.0 license (CC BY-SA 2.0). As such, we have the credited OpenStreetMap as and its contributors as required in the “Study design and data collection” subsection of the Materials and Methods (Page 7, Line 129-130).

Reviewer #1

1. The title of the study suggests a description of animal rabies cases. However, the study content suggests that this a study of knowledge about rabies among people who own certain animals. I strongly suggest the authors reframe the title of the study.

- Thank you for your suggestion. We agree with your comment and have changed the title to more precisely reflect the study. The new title includes a subtitle that explicitly calls attention to the knowledge, attitudes, and practices of the people in the region. The new title now reads “Descriptive study of cattle and dog rabies cases in the Sheki-Zagatala region of Azerbaijan (2015-2016): Knowledge, attitudes, and practices of people towards rabies.”

2. How many of the 84 animal rabies cases in two years (2015-2016) were cattle and dogs who died of rabies cases before Aug 14, 2016? If this number is >20, why have the authors included only 20 cases in this study?

- Thank you for bringing this point up. We were mainly interested in Sheki-Zagatala region as there was a disproportionate number of animals affected in this region. Indeed, from Jan 1, 2015 through Aug 14, 2016, there were 9 human rabies cases and 66 animal rabies cases throughout Azerbaijan. This includes 26 farm animals (large and small ruminants), 33 dogs, and 7 wild animals (5 jackals and 2 wolves). In this current study we investigated 14 of the animals that were from this region and encountered 6 more previously unreported cases during our survey. Ultimately, we would like to perform a lager case-control study to identify risk factors that may be specific to this region. (page 4, lines 77-79)

3. Line 76 - Authors should mention "This study describes the epidemiological profile of animal rabies cases …….”. Why does the knowledge of owners about rabies not find mention in the study objectives???

- Thank you for mentioning the objectives. We corrected the sentence and now the last paragraph in the introduction clearly describes the study objectives (page 5, lines 90-92)

4. In “Study design and data collection” section the preferable order of paragraphs seems Para 2, then Para 1, and then Para 3.

- Thank you for the advice. We completely agree with this point and have changed the order of paragraphs in “Study design and data collection” section.

5. How were the non-cases selected? What is the rationale of interviewing non-cases? What information do they add to this study? Why did the authors choose 80 non-cases?

- Thank you for giving us a chance to discuss and clarify this point. We selected animal owners from the same communities as those with animals that were infected with rabies. We wanted to know if there were any common factors that might reduce and/or increase the risk of infection. We would eventually like to conduct a larger case-control study of all animal cases in Azerbaijan, outside of Sheki-Zagatala, as compared to those in this specific region. We selected a ration of 1:4 (case owners to non-case owners) as a way to better engage the community and help set future design parameters.

6. In “GPS Coordinates” section, the sentence seems incomplete. An “of” seems missing after “locations”.

- Thank you for catching the mistake. The sentence has been rephrased (Page 7, line 129-130).

7. In Table 1, it appears that only 4+1+1 = 6 dogs went out of the backyard, while in Line 120, the authors mention “8 were permitted to roam untethered in the community.”

- Thank you for the comment. We have corrected the mistake. The right number is 6. This is the number of case dogs that reportedly roamed untethered in the community (page 8, line 143).

8. What is meant by “Animal identification” in Table 1?

- Thank you for asking and giving us a chance to clarify. This is due to our translation. We changed it to “Animal registration number”.

9. In Table 1, five dogs and seven cattle were reported to be bitten by ‘any animal’, but the biting animal adds to only 2 (1+1) for dogs and 6 (3+3) for cattle. Why is the information missing for others?

- Thank you for catching this. To avoid a further confusion on readers, we added extra line in Table 1 under the question “Bitten by which animal”. Also, we added clarification on this point in page 11, lines 158-163 (immediately after Table 1) that helps to understand how those animals identified as being bitten.

10. In Line 189, the authors mention that “residents in the Sheki-Zagatala region lack specific knowledge on rabies transmission, risk factors, and perceived severity of the disease”. There are several problems with this sentence. Firstly, the authors have not enquired about “severity of the disease” from the participants. Secondly, the very next sentence is “ …… correctly indicated as a means of transmission of the disease by a majority …….”. And more importantly, how is it justified to generalize the findings from 20+80=100 persons (method of selection unknown) to the entire population of the region?

- Thank you for bringing up this point. We changed the “severity” to “seriousness” and by this meant that sometimes the animal owners don’t perceive rabies as a serious disease by not vaccinating their dogs, or not following what happened to their rabid animal. The sentence finds its clarification in farther paragraphs in discussion section.
- Thank you for helping us to understand that the generalization of findings from 100 persons to the entire population would be wrong and the numbers too small to be representative (page 19, lines 218-219).

11. Line 196: Recheck the first sentence. Did you intend to write “for” instead of “as”?

- Thank you for catching up the mistake. It is corrected (page 19, line 226)

12. Line 206: The sentence “The locations of pastures ……” seems incomplete.

- Thank you for catching up the mistake. We completed the sentence (page 20, line 236).

13. The “Discussion” section must contain a paragraph discussing the strengths and limitations of the study.

- Thank you for helping us understand that we miss an important section. Based on this comment we added the paragraph describing the strengths and limitations of the study into the discussion section (page 21, lines 272-278).

Reviewer #2

Major comments:

1. Look at the result when comparing the 20 cases and 80 no-case owners, it seems that the case owners have more knowledge, good attitude, and practices regarding rabies than the no-case owners. To confirm this observation, Statistical analysis should be applied to this data for comparison, which is not the case in this study; authors should think about it to give strong confidence and significance to the findings. In that case, a column can be created for each table to include the statistical values.

- Thank you for bringing this point up. Ultimately, we would like to conduct a more robust case-control study to look identify potential risk factors for rabies in this region. However, for this preliminary observational study, we were more interested in the general knowledge, attitudes and practices of people in this region. We do not believe that a statistical analysis on this group would be very meaningful as this includes a mix of dog and cattle owners and therefore is more likely to add confounding. We have thus decided to combine the data to provide a baseline assessment at this time.

Miner comments:

1. Line 60-63: for the rabies situation in Azerbaijan some control measures such as vaccination was implemented and 465,000 animals were vaccinated between 2015 and 2016; 1,217,559 from 2012 to 2016. However, the total number of the animal population is not provided that can give an estimation of the vaccination coverage; and this data can be included in the discussion part to show whether the vaccination is still not effective to prevent rabies before speculating on other factors.

- Thank you for bringing up this problem. Unfortunately, we were not able to get total number of the animal population in Azerbaijan at the time of study conducted, which could help us to discuss the vaccine coverage and would show the real situation with dog vaccination program in Azerbaijan (page 4, lines 74-75).

2. Line 102-104: the purpose of the electronic questionnaire is not clearly explained and data was not further exploited in the study (face to face interview was carried out); it can be deleted instead because not informative.

- Thank you for mentioning the purpose of the electronic questionnaire. The aim of including e-questionnaire was to evaluate effectiveness of switching to paperless surveys/studies. Found out that in high latitude villages it was not possible to implement e-questionnaire because of weak internet. Based on this comment, the section about e- questionnaire was removed (page 7, lines 123-125).

3. Line 106, the GPS coordinates can be included in the study design and data collection section, not as a subtitle.

- Thank you for the suggestion. The subtitle was removed, and the line was included to the study design section (page 7, line 127).

4. Line 121-122: the respondents claimed that 3 vaccinated dogs were bitten but got infected and died, this information is not discussed in the study; this should be discussed as vaccine failure or incorrect information from the owners or died from other cause than rabies, ...

- Thank you for touching the issue. It helped us to understand that we have missed an important section in discussion. The problem with vaccinated dogs who died because of rabies was discussed as an incorrect information from the owners in page 20 lines 241-255. Answers about animal vaccinations from owners, did not match the records of the veterinarians. We conclude that the owners must have been mistaken or forgot.

5. Line 128-130: It is stated that among 11 cattle, 3 were vaccinated against rabies for post-exposure; and 7 were bitten and died. Could the authors clarify if dead cattle include 3 vaccinated ones? If so, this should be discussed in the same way as for vaccinated dogs that died "vaccine failure or wrong information from owners, …”.

- Thank you for the advice. Based on it, we continued discussing the problem with vaccinated cattle who died of rabies in page 20 lines 256-264.

6. Given that the rabies cases in this study are based on clinical diagnosis, authors should briefly describe the clinical manifestations of rabies in different animal species that will support the results as provided in line 142-144 and figure 2. Description of furious and paralytic rabies can help readers to understand the situation, especially in line 161 where cattle bite humans.

- Thank you for the recommendation. Three new references added to help describe the difference between furious and paralytic rabies (page 4 lines 55-59). I) Tepsumethanon V, Likitsuntonwong W, Thorner PS, Shuangshoti S. Dogs that develop rabies post-vaccination usually manifest the paralytic subtype. Preventive Veterinary Medicine. 2016;131:64–66, II) Schildecker S, Millien M, Blanton JD, Boone J, Emery A, Ludder F, et al. Dog ecology and barriers to canine rabies control in the Republic of Haiti, 2014-2015. Transboundary and Emerging Diseases. 2017;64(5):1433-1442 and III) Shuangshoti S, Thorner PS, Teerapakpinyo C, Thepa N, Phukpattaranont P, Intarut N, et al. Intracellular spread of rabies virus is reduced in the paralytic form of canine rabies compared to the furious form. PLOS Neglected Tropical Diseases. 2016;10(6):e0004748. doi: 10.1371/journal.pntd.0004748” were added to the manuscript. (page

7. Line 161 and Q3 in table 3: is cattle bite happened frequently in rabid cases? In figure 2, the aggressively against humans was reported only in 6 animals, are these cattle or dogs? Could you clarify this point?

- Thank you for catching this. Based on this comment we included updated Figure 2 which shows the symptoms separately for rabid cattle and rabid dogs. In 4 out of 11 rabid cattle observed aggressiveness against owners while the same symptom was observed in 2 out of 9 rabid dogs.

8. Line 196-198: please include reference(s) for this statement about the common source and cause of rabies in Azerbaijan.

- Thank you for catching the missing reference. The reference for the statement was added (page 19, line 228)

9. Line 206: remove by before the reference “ …was reported as a risk factor [15]"

- Thank you for catching the mistake. The sentence is corrected and completed (page 20, line 236).

10. Line 215: no information or data is revealing that rabies is underreported in the study, could you describe this point?

- Thank you for asking and giving us a chance to clarify this point. In this current study we investigated 14 animals from the Sheki-Zagatala region that were confirmed positive for rabies by the Republican Veterinary Laboratory. Additional 6 cases (2 cattle and 4 dogs) encountered as new case during the study through epidemiological links, symptoms of death and date of onset of disease. These 6 cases have not been reported to the RVL. Based on these unreported cases in the small survey we conducted, we believe there are likely to be more unreported cases in Sheki-Zagatala, and in the rest of Azerbaijan.

We believe that the manuscript in now suitable for publication in PLOS ONE.

**Aytan Garayusifova**

MPH student at the University of Washington

[ayten.yusifzade@gmail.com](mailto:ayten.yusifzade@gmail.com)

On behalf of all authors.
